# Ventrículo derecho de doble cámara en un paciente con miocardiopatía hipertrófica no obstructiva. Reporte de caso

**DOI:** 10.47487/apcyccv.v6i1.440

**Published:** 2025-02-12

**Authors:** Juan María Iroulart, Joaquín Ferrero, Rocío Blanco, Roshan Licht, Ana Miceli, María Natalia Pellegrini, Diego Pérez de Arenaza, Rodolfo Pizarro

**Affiliations:** 1 Servicio de Cardiología, Hospital Italiano de Buenos Aires, Ciudad Autónoma de Buenos Aires, Argentina. Servicio de Cardiología Hospital Italiano de Buenos Aires Ciudad Autónoma de Buenos Aires Argentina

**Keywords:** Cardiomiopatía Hipertrófica, Ventrículos Cardíacos, Diagnóstico por Imagen, Cardiomyopathy Hypertrophic, Heart Ventricles, Diagnostic Imaging

## Abstract

Se presenta el caso de un paciente masculino de 42 años de edad con antecedente de trasplante bipulmonar con disfunción crónica del injerto. El paciente acudió a la central de emergencias de adultos por cuadro de insuficiencia cardíaca aguda. Durante su estancia en guardia y en seguimiento ambulatorio, mediante las multiimágenes cardíacas se diagnosticó ventrículo derecho de doble cámara con miocardiopatía hipertrófica asociada. Debido a su enfermedad pulmonar avanzada y escasa adherencia al seguimiento médico, se decidió no realizar un re-trasplante pulmonar. Se optimizó su medicación inmunosupresora y se añadieron betabloqueantes como parte del tratamiento de la obstrucción dinámica intraventricular derecha. Además, se derivó a rehabilitación pulmonar, mostrando actualmente una evolución parcialmente favorable en clase funcional II.

## Abstract (en)

We present the case of a 42-year-old male patient with a history of bilateral lung transplantation and chronic graft dysfunction. The patient presented to the adult emergency department due to acute heart failure. During his stay in the emergency room and in outpatient follow-up, cardiac multi-imaging led to the diagnosis of double-chambered right ventricle with associated hypertrophic cardiomyopathy. Given the presence of advanced lung disease and poor adherence to immunosuppressant medication as well as clinical follow-ups, the patient was deemed unsuitable for re-lung transplantation. The optimization of his immunosuppressive medication was prioritized, and beta-blockers were added as part of the treatment for dynamic right ventricular outflow obstruction. He was referred to pulmonary rehabilitation, currently showing a partially favorable evolution to functional class II.

## Introducción

El ventrículo derecho (VD) de doble cámara es un defecto cardíaco infrecuente. Su etiología es congénita en la mayoría de los casos, constituyendo entre el 0,5 y el 2% de todas las cardiopatías congénitas, aunque existen escasos reportes de la etiología adquirida ^1^. Puede ser causado por bandas musculares anómalas, hipertrofia trabecular o una banda moderadora aberrante ^1^^,^^2^.

Numerosos ensayos han reportado su asociación con otras anomalías cardíacas (especialmente comunicaciones interventriculares) y extracardíacas ^3^. Sin embargo, solo algunos reportes de casos describieron la coexistencia del VD de doble cámara en pacientes con miocardiopatía hipertrófica (MCH) ^4^^,^^5^. A continuación describimos un paciente en quien se diagnosticó MCH y VD de doble cámara en la edad adulta.

## Reporte de caso

Se presenta el caso de un paciente masculino de 42 años de edad sin factores de riesgo cardiovascular. Presenta como antecedentes relevantes un familiar de primer grado (padre) con MCH. En cuanto a sus antecedentes personales, se destaca un trasplante bipulmonar por bronquiectasias adquiridas en el año 2020, con bronquiolitis obliterante y rechazo del injerto pulmonar a los 3 meses por inadecuada adherencia a la inmunosupresión, que culmina en disfunción crónica del injerto. 

El paciente acude a la central de emergencias de adultos por disnea en clase funcional III, sin ortopnea ni disnea paroxística nocturna. Además síntomas de apneas e hipersomnolencia diurna y síntomas de obstrucción traqueal dinámica como secuela posquirúrgica. 

**Figura 1:**
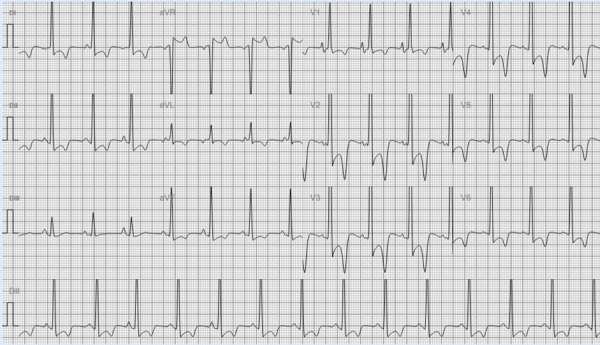
Electrocardiograma en ritmo sinusal a 90 latidos por minuto. Onda P pulmonar. QRS angosto con rotación horaria y marcados trastornos de la repolarización de manera difusa.

**Figura 2:**
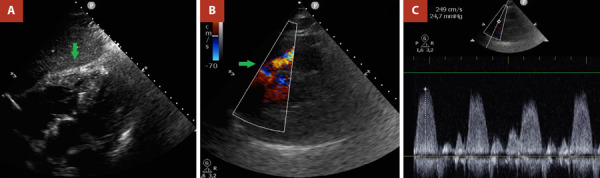
Ecocardiograma transtorácico con ventana acústica subóptima desde **(A)** vista subxifoidea mostrando marcada hipertrofia medioapical de la pared libre de VD (flecha verde) y **(B)** eje corto de grandes vasos con aceleración medioventricular derecha (flecha verde) con **(C)** gradiente intraventricular del VD.

Al examen físico se presentaba normotenso, taquicárdico, eupneico con buena mecánica ventilatoria. Se auscultaban rales crepitantes tipo velcro bibasales y un soplo sistólico en foco pulmonar 2/6. Además, presentaba edemas en miembros inferiores 2/6. En primera instancia se realizó un electrocardiograma que evidenció un ritmo sinusal con signos de hipertrofia biventricular y trastornos marcados de la repolarización (Figura 1). Por este resultado se decide realizar un ecocardiograma transtorácico (ETT) que denota una severa hipertrofia del ventrículo izquierdo (VI). Además de hipertrofia del VD con obliteración de segmentos apicales y un gradiente intraventricular de 24 mmHg (Figura 2). Asimismo, se evidenció un reflujo tricúspideo leve, dilatación de aurícula derecha (AD) con gradiente VD-AD de 41 mmHg y fracción de eyección biventricular levemente deprimida en relación a hipocinesia global.

**Figura 3:**
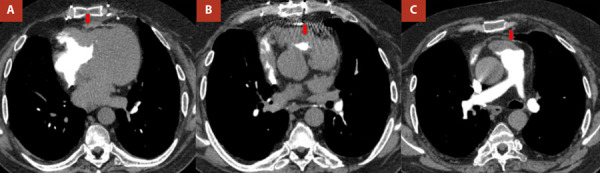
Angiotomografía de tórax que evidencia ausencia de relleno sugestiva de marcada hipertrofia de la pared libre ventricular derecha **(A, flecha)** con estrechamiento del tracto de salida en cortes hacia cefálico llegando a una mínima luz **(B, flecha)** hacia la arteria pulmonar **(C, flecha)**.

Se realizó una angiotomografía de tórax para descartar tromboembolismo pulmonar, la cual fue negativa, aunque reflejó una hipertrofia biventricular notable (Figura 3). Con la interpretación diagnóstica de insuficiencia cardíaca congestiva, recibió 20 mg de furosemida por vía intravenosa y, tras buena respuesta, se decidió su egreso hospitalario para evaluación ambulatoria adicional de su cardiopatía estructural.

**Figura 4:**
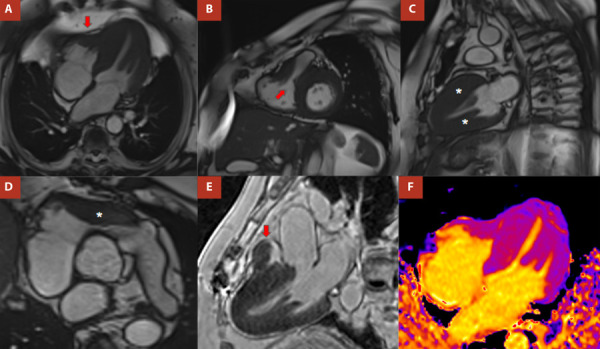
Resonancia Magnética Cardiaca. **A:** vista de cuatro cámaras que evidencia la obliteración de segmentos apicales del VD (flecha) e hipertrofia biventricular. **B:** sección sagital a través del estrechamiento subvalvular a nivel del tracto de salida del VD (flecha) producido por la hipertrofia del tabique interventricular y de la pared libre del VD. **C:** Vista de dos cámaras de VI que expone la notoria hipertrofia septal y de la pared posterior (asteriscos). **D:** eje corto de grandes vasos que denota la hipertrofia en el tracto de salida de ventrículo derecho (asterisco) con obliteración de este. **E:** imagen de realce tardío de gadolinio (RTG) con patrón tenue intramiocárdico difuso con resalto de la hipertrofia de ventrículo derecho (flecha). **F:** secuencia de T1 mapping nativo con valor miocárdico de 1123.0 ms (aumentado) compatible con fibrosis difusa.

Como parte del estudio anatómico se realizó una resonancia magnética cardiaca (RMC) (Figura 4) que mostró signos de MCH izquierda no obstructiva con compromiso de VD a predominio de tracto de salida y de los segmentos apicales (máximo espesor de 17 mm en TSVD) que ocasionaba una estenosis subpulmonar y una obliteración sistólica de dicha cavidad, además de deterioro leve de la FE VD (51%). La masa del VI se encontró severamente incrementada (153 g/m^2^; máximo espesor parietal a nivel anteromedial de 24 mm) con fracción de eyección normal. En las imágenes después de la inyección intravenosa del gadolinio se observó realce con patrón intramiocárdico difuso tenue en los segmentos anteroseptal medial, inferoseptal medial, anteroapical, septal apical, inferoapical, lateral apical, apical estricto y en la pared libre de ventrículo derecho. Este último hallazgo permitió descartar enfermedades subendocárdicas como la fibrosis endomiocárdica. Las secuencias de T1 *mapping* nativo de 1123 milisegundos fueron compatibles con la presencia de fibrosis intersticial difusa, mientras que el volumen extracelular y el T2 *mapping* estaban conservados.

La prueba de evaluación de la función respiratoria en ejercicio mostró un defecto ventilatorio mixto a predominio obstructivo severo, con capacidad vital forzada disminuida en forma severa, compatible con difusión crónica del injerto pulmonar.

Dada la presencia de enfermedad pulmonar avanzada y debido a la mala adherencia al consumo de inmunosupresores así como a los controles clínicos, el paciente fue desestimado como candidato a re-trasplante pulmonar. Se priorizó la optimización de su medicación inmunosupresora y se añadieron betabloqueantes como parte del tratamiento de la obstrucción dinámica intraventricular derecha; se derivó a rehabilitación pulmonar, mostrando actualmente una evolución parcialmente favorable en clase funcional II.

## Discusión

El compromiso del VD en la MCH es poco común (aproximadamente 15% de los pacientes) y la enfermedad predominantemente o aislada del VD es aún más infrecuente ^6^. Su mecanismo de formación es congénito en la mayoría de los casos, constituyendo entre el 0,5 y el 2% de todas las cardiopatías congénitas, aunque también puede ser adquirido, como el compromiso de cavidades derechas en una miocardiopatía hipertrófica ^1^. En este trastorno existe un gradiente intraventricular derecho superior a 20 mmHg entre una cámara proximal de alta presión cercana a la válvula tricúspide y una cámara distal de baja presión adyacente a la válvula pulmonar. El defecto puede ser causado por bandas musculares anómalas, hipertrofia trabecular o una banda moderadora aberrante ^1^^,^^2^. Los hallazgos histológicos parecen ser similares a los del compromiso del VI, lo cual sugiere una patogénesis similar, aunque la obstrucción del TSVD del tipo doble cámara es más infrecuente y puede resultar en síntomas más graves y de difícil tratamiento ^7^. Si bien la fisiopatología no está claramente establecida, se cree que la obstrucción del TSVD y la reducción del gasto cardiaco (por compromiso del llenado del VD) podrían contribuir al empeoramiento de los síntomas. En el caso de nuestro paciente, su asociación con miocardiopatía hipertrófica izquierda y la ausencia de otros defectos cardíacos congénitos sugieren una etiología adquirida, lo que lo incluye en un reducido grupo de reportes en la bibliografía.

Los síntomas producidos por esta condición son similares a los de otras patologías cardiovasculares, siendo la disnea y la intolerancia al ejercicio los dos más frecuentes ^8^. Estos evolucionan a tolerancia mínima o disnea incluso en reposo a medida que el gradiente obstructivo del VD aumenta. En nuestro caso, el paciente presentaba disfunción crónica del injerto pulmonar, por lo cual los síntomas respiratorios se encontraban exacerbados. A pesar de esto, se destaca su presentación en la adultez como un hallazgo infrecuente.

La ecocardiografía y el cateterismo cardíaco son útiles para evaluar la hipertrofia ventricular, las presiones diastólicas y los gradientes del TSVD, respectivamente. La RMC es actualmente el mejor método diagnóstico, ya que proporciona imágenes de mayor calidad para una evaluación minuciosa de la distribución biventricular de la hipertrofia y ayuda a excluir otras causas de anomalías ventriculares ^9^. Asimismo, contribuye al diagnóstico diferencial con la presencia de otras causas de obliteración de segmentos apicales del VD como pueden ser la endomiocardiofibrosis y los tumores cardíacos.

Aunque se desconoce cuál es el tratamiento adecuado, los pacientes sintomáticos han sido tratados con betabloqueantes y bloqueadores de los canales de calcio, mostrando una disminución de los síntomas y de los gradientes intraventriculares de forma variable ^10^. En segundo lugar, la estratificación del riesgo de MSC es mandatoria en pacientes con MCH; sin embargo, los *scores* de riesgo no contemplan el compromiso de VD como una variable a jerarquizar y desconocemos si esto puede tener un peso relativo mayor que la ausencia de compromiso de VD ^11^^,^^12^. Por último, existen casos de resolución quirúrgica de la doble cámara ventricular derecha mediante miectomía, con o sin miocardiopatía hipertrófica asociada ^13^. Esta opción no fue considerada en nuestro paciente por el elevado riesgo quirúrgico y las comorbilidades asociadas, pero hubiera sido la mejor opción debido al severo compromiso obstructivo dinámico.

En conclusión, la asociación entre la doble cámara de ventrículo derecho y la miocardiopatía hipertrófica es infrecuente. Esta requiere de una evaluación cardiovascular exhaustiva utilizando los diferentes métodos de imágenes para arribar al diagnóstico. De esta manera, se presentó un caso clínico de una asociación infrecuente y con presentación clínica en la edad adulta, lo cual lo convierte en un hallazgo de relevancia. Esto resalta la importancia de que los cardiólogos de adultos reconozcan la patología a pesar de los escasos reportes a partir de la segunda década de la vida.

## Fuente de financiamiento:

Autofinanciado
